# “Dear Doctor, greetings of the day!”: A 1-year observational study of presumed predatory journal invitations

**DOI:** 10.1186/s40510-023-00471-6

**Published:** 2023-07-03

**Authors:** Christos Livas, Konstantina Delli

**Affiliations:** 1 Division of Orthodontics, Dental Clinics Zwolle, Stationsweg 5, 8011 CZ Zwolle, The Netherlands; 2 Department of Oral and Maxillofacial Surgery, University Medical Center Groningen, University of Groningen, Groningen, The Netherlands

**Keywords:** Predatory journals, Open access, Ethics, Academics

## Abstract

**Background:**

This study aimed at investigating the predatory publishing phenomenon in orthodontics by analyzing the content of unsolicited e-mail invitations received within 12 months.

**Methods:**

All electronic invitations for manuscript submission, review and editorial membership received between 1 October 2021 and 30 September 2022 were collected from an orthodontist’s inbox. The following data were recorded for each e-mail: date, journal title and origin, requested contribution, e-mail language, relevance to the researcher’s discipline, journal characteristics (claimed metrics, editorial services, article types accepted, and publication fees), journal/publisher contact information and online presence. Journal/Publisher legitimacy and publishing standards were evaluated by listing in the Beall’s list of potential predatory journals and publishers, the Predatory Reports of Cabell’s Scholarly Analytics, and the Directory of Open Access Journals.

**Results:**

A total of 875 e-mail invitations deriving from 256 journals were retrieved within the observation period, with most of them soliciting article submissions. More than 76% of the solicitations originated from journals and publishers included in the blocklists used in the study. Salient features of predatory journals like flattering language, abundant grammatical errors, unclear publication charges and wide variety of article types and topics accepted for publication were confirmed for the examined journals/publishers.

**Conclusions:**

Nearly 8 out of 10 unsolicited e-mail invitations sent to orthodontists for scholarly contribution may be related to journals suspicious for publishing malpractices and suboptimal standards. Excessive flattering language, grammatical errors, broad range of submissions, and incomplete journal contact information were commonly encountered findings. Researchers in orthodontics should be alert to the unethical policies of illegitimate journals and their harmful consequences on the scientific literature.

## Introduction

The rise of the open access (OA) movement, intrinsically linked to the development of the Internet in the 1990s, has radically transformed scientific publishing operations [1]. By enabling free online availability of published research articles, OA journals appeared to offer higher visibility compared to those of traditional journals, faster dissemination as a result of generally shorter publication process, and increased likelihood of citation [2]. In the typical OA business model, article publication or processing charges (APCs) are shifted from the reader to the author to enable free and immediate access to the article content [3].

The OA publishing model is commonly manipulated by predatory journals promoting themselves as legitimate OA journals, seeking profit through claimed APCs [4]. The modus operandi of predatory journals, also called fraudulent, deceptive or pseudo-journals [5], is primarily characterized by false or misleading information, deviation from essential publishing standards, lack of transparency, and aggressive e-mail solicitations [6]. The number of predatory publications is currently estimated to exceed 15,000 titles overtaking and growing faster than the legitimate or mainstream journals [7].

The exponential growth of the predatory publishing puts science at risk in several ways. Due to the lack of a rigorous quality assurance mechanism, that is peer-review, flawed and unvetted evidence published in pseudo-journals may influence laypersons’ beliefs and medical decision making [8, 9]. Similar to this, inclusion of poorly conducted studies in systematic reviews may undermine the validity of conclusions with devastating effects on patient care [4]. As questionable-quality articles are also getting cited by non-experts, causing “citation contamination” [10], the reach of genuine, legitimate research is compromised. Given indexing and perpetual content preservation are rare, the content of predatory journals cannot be easily retrieved from scholarly online databases limiting the impact of publicly funded research. This implies that patients or animals involved in the studies, research manpower and taxpayers’ money might have been wasted [4]. For an organization of 100,000 email users with an annual salary of $100,000, receiving 6 spam e-mails per day like those from illegitimate publishers, the cost in terms of information technology management and lost work hours has been estimated to exceed $16 million per year [11]. As publication productivity is getting nowadays rewarded in promotion and tenure decisions in academia [12], young scholars are placed under constant pressure of career advancement deadlines to establish a substantial publication track record and curriculum vitae over a short period of time [13]. Not surprisingly, eighty percent of research grant awardees reported to regularly spend time to read and sort predatory publishing e-mails [14]. Authors and reviewers contributing to predatory journals share the same characteristics; a young academic age, a short list of publications, and origin from low-income and lower middle-income countries [15]. Nonetheless, not only early-career researchers but also established academicians may fall victims to predatory journal practices [16].

Numerous blocklists and allowlists have been developed to help scholars to discern presumed predatory and legitimate publications, respectively. However, those lists tend to focus on easily identifiable criteria, while parameters like review process and other editorial services are not comprehensively reviewed. This discrepancy allows misclassification of some journals or others to operate in “a grey zone between fraud and legitimacy” [17].

While biomedicine was ranked as one of most active disciplines in generating predatory articles [18], little has been published so far on the extent and patterns of predatory publishing solicitation in dentistry [19–21]. Six hundred forty-two out of 647 journal titles that sent an invitation to an oral epidemiologist in 12 months for manuscript submission met some of the criteria to be presumed predatory or of very low quality [20]. Analysis of all unsolicited invitations for manuscript submission received by a faculty periodontist within 1 year showed that 88.54% of the e-mails derived from journals classified as predatory [21]. Seeing that no similar research has been conducted to date in orthodontics, this study aimed to describe the prevalence of predatory publishing and the characteristics of e-mail invitations for manuscript submission, review and editorial board membership sent to a single orthodontist within 12 months.

## Materials and methods

All unsolicited invitations received from presumed predatory journals were prospectively collected between 1 October 2021 and 30 September 2022 from the first author’s e-mail address, which had been used in the past for correspondence with journals during the publication process. The recipient is a mid-career researcher in orthodontics with faculty and research appointments at universities and oral healthcare settings. To avoid interference with journals’ solicitation strategies and, likely, missing data, the recipient neither replied to any of the invitations nor requested to unsubscribe during the whole observation period [22]. Both inbox and spam folders were screened for solicitations for scholarly contributions by 2 reviewers, and disagreements were resolved by consensus. Following similar research [22], only invitations for manuscript submission, review and journal’s editorial board membership were collected. Thus, e-mails irrelevant to the purposes of the study like solicitations for book or book chapter submission, certified short-term fellowships, advertisements, promotion of new products, editing/proofreading services, grant application consultants, conferences, and webinars were excluded.

The following data were retrieved by the 2 reviewers on the basis of consensus from the e-mails meeting the inclusion criteria: date, journal and publisher titles, sender’s name and position in the journal/publisher, type of requested contribution (i.e., manuscript, review or participation in the journal’s editorial board), e-mail origin, salutation, language (i.e., spelling or grammar errors, flattery, deadline to respond, appealing title) relevance to the researcher’s discipline (i.e., previous publication cited, invitation related to author's specialty), journal characteristics (i.e., IF, ISSN, claim for editorial services, broad range of documents accepted, discounted publication fee) journal/publisher contact information (i.e., phone number, street address), and online presence (i.e., website availability, online verifiability, external links provided, “unsubscribe” mechanism, disclaimer presence) [11, 22]. Whenever an e-mail contained invitations from multiple journals, each invitation was counted individually. Publishers were identified by screening the journal websites, where available, and were verified by 5 freely available databases using the following links: https://www.citefactor.org/; https://journals.indexcopernicus.com; https://journalseeker.researchbib.com/; https://www.rootindexing.com/; https://www.scilit.net/. If no publishing company could be traced back, respective journals were deemed standalone.

Journal/Publisher legitimacy was determined by inclusion or not in the original and updated Beall’s list of potential predatory journals and publishers (https://beallslist.net/), and the Predatory Reports of Cabell’s Scholarly Analytics (https://www2.cabells.com/). Furthermore, journal publishing standards were evaluated by means of listing in the Directory of Open Access Journals (DOAJ; https://doaj.org). Classification of journals as presumed predatory or illegitimate was based on the inclusion of the journals or respective publishers in Beall’s or Cabell’s blocklists.

Invitation type and predatory journal classification were examined for the total of the e-mail invitation received during the observation period. After removing duplicates, invitation and journal characteristics were recorded in numbers and percentages. All collected data were imported into a Microsoft Excel worksheet (Microsoft Corporation, Redmond, WA, USA) for further analysis.

## Results

**Fig. 1:**
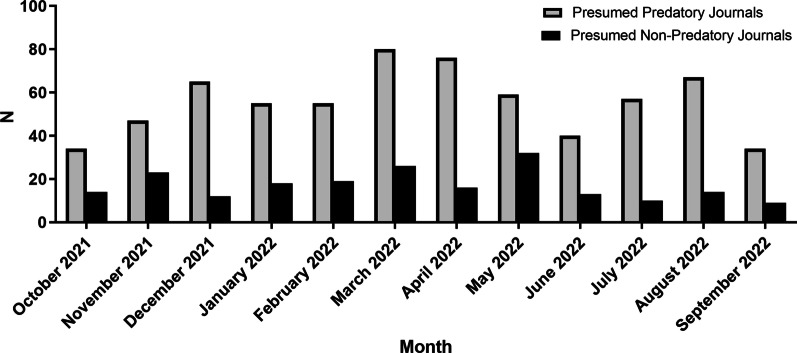
Distribution per month and journal classification of all e-mail invitations received during the observation period

A total of 875 eligible electronic invitations deriving from 256 journals were collected during the 1-year observation period. Out of the solicitations received, 831 called for article submission, 17 for editorial board membership, 3 for article review, and the rest for a combination of contributions (Table 1). The largest number of invitations across months, that is 106, was recorded in March 2022, while the maximum number of invitations received on a single day was reached on July 16, 2022. Six hundred sixty-nine invitations, i.e., 76.46%, originated from journals and publishers included in Beall’s lists and the Predatory Reports of Cabell’s Scholarly Analytics (Table 1). Nine e-mail solicitations were sent by journals listed in DOAJ. The monthly distribution of invitations per predatory classification is illustrated in Figs. 1.

**Table 1:** Distribution of the total of e-mail invitations received within 12 months per type of requested contribution and predatory journal classification

Contribution type	N	%
Article submission	831	94.97
Editorial board membership	17	1.94
Article review	3	0.34
Article submission/Editorial board membership	12	1.37
Article submission/Editorial board membership/Article review	2	0.24
Editorial board membership/Article review	10	1.14
Total	875	100.00

Journal classification	N	%
Presumed predatory journals	669	76.46
Presumed non-predatory journals	206	23.54
Total	875	100.00

Twenty-seven journals were considered standalone journals, while the rest derived from 89 unique publishers overseeing the publication of 1–24 journals. Details about the included information in the examined e-mail invitations are presented in Table 2. Biomedical journals represented 83.59% of the e-mail senders, with dental journals invitations, related from a broader perspective to the author’s specialty, appearing 74 times. A journal devoted to dentistry and oral biology sent up to 12 e-mail solicitations per month, while 4 solicitations were forwarded on a single day by a biogeneric journal.

**Table 2:** Information included in the 256 unique e-mail invitations examined in the study

Included information	N	%
Paid e-mail service	243	94.92
Salutation including name	167	65.23
Generic salutation	65	25.39
IF	66	25.78
ISSN	80	31.25
Claim for editorial services	133	51.95
Broad range of accepted article types	224	87.50
Discounted APCs	40	15.63
Website availability	235	91.80
Online verifiability	10	3.91
External links provided	145	56.64
Unsubscribe mechanism	151	58.98
Disclaimer presence	49	19.14
Spelling or grammar errors	193	75.39
Flattery	107	41.80
Submission deadline	145	56.64
Appealing journal title	63	24.61
Contact phone number	21	8.2
Street address	52	20.31
Previous publication cited	14	5.47
Relevance to invitee’s specialty	75	29.30

**Fig. 2:**
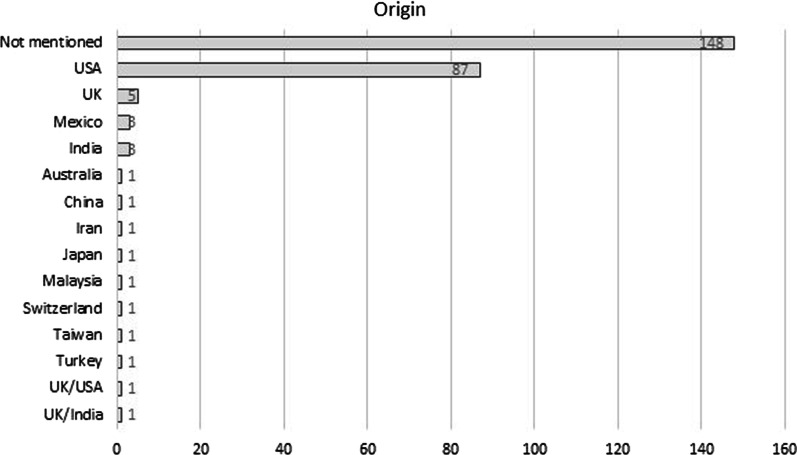
Distribution of the 256 unique e-mail invitations examined in the study per origin

Thirteen e-mail invitations, i.e., 5.08% of the total, were sent via free e-mail providers, either Yahoo mail or Gmail. Journal origin was disclosed in 108 invitations with USA being the most frequently mentioned country of origin (Fig. 2). Contact phone numbers and street addresses of the journal/publishers were rarely cited, i.e., merely in 8.20% and 20.31% of the e-mail solicitations (Table 2).

The invitee was addressed in the e-mail introduction by his name in 167 invitations, i.e., 65.23%, whereas a generic salutation like “Dear/Esteemed/Respected Author/Doctor/Professor/Researcher, Greetings of the day!” was used in 25.39% of the cases (Table 2). Recipient’s name or salutation was missing in the remaining 24 email invitations.

Journal Impact Factors (IFs) and International Standard Serial Numbers (ISSNs) were mentioned in 66 and 80 e-mail invitations, respectively. Provision of editorial services, e.g., peer-review, assignment of Digital Object Identifier (DOI), plagiarism control and indexing by scientific databases was mentioned in slightly over half of the journals, i.e., 51.95% of the analyzed publications. The vast majority of the journals inviting manuscript submission, i.e., 224 out of 243, welcomed for publication a broad range of article types, including original research, reviews, case reports, clinical images, editorials, commentaries, conference proceedings. Substantially low APCs, ranging between $20–399, were offered by the examined journals. Confusing publication fee benefits and discounts were granted under conditions by 40 journals (Table 2). Specifically, attractive publication charges were offered as full or partial APC waiver due to the pandemic crisis, in case of authors originating from middle- or low-income countries or submission completed within the given short deadline.

Regarding journal online presence, websites were available in 235 journals, but only 10 journals appeared to have articles indexed in PubMed. “Unsubscribe” mechanisms and disclaimers were included in 153 and 49 journals, respectively (Table 2). While external links were provided in 145 e-mails, 95 of those links were broken and directed to messages like “Site could not be reached”, “Account suspended” or “Error; page not found”.

**Fig. 3:**
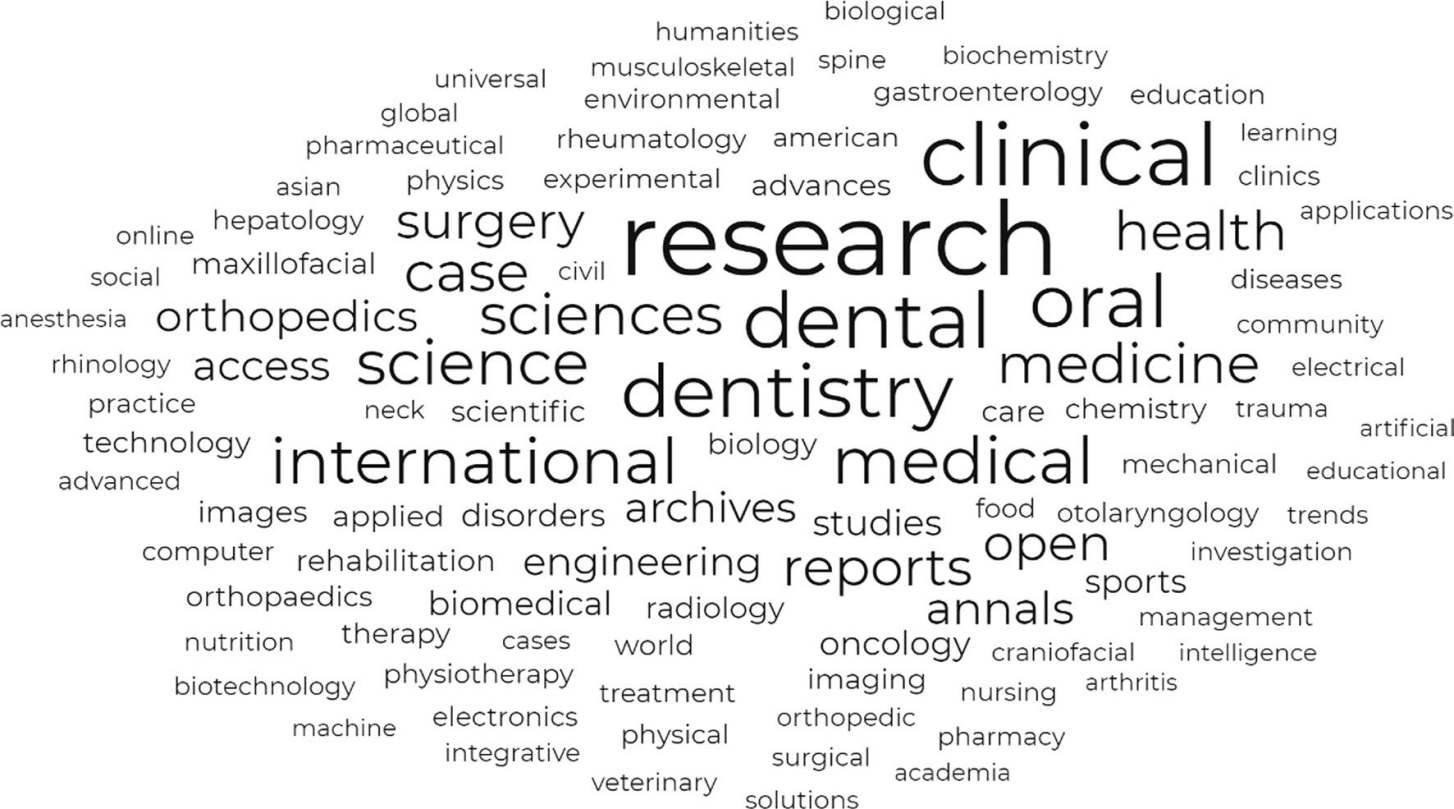
Word frequency in the investigated journal titles. The world cloud was generated using Free World Cloud Generator; https://www.freewordcloudgenerator.com/

Flattering or excessive flattering language was used in 107 e-mails, while spelling and grammatical errors were found in more than 75% of the solicitations. Characteristic examples of flattering language used in the e-mails are displayed in Table 3. A deadline for manuscript submission was cited in 145 out of 243 related solicitations (Table 2). “Research” and “clinical” were the most common words, appearing in 53 and 42 titles, respectively, followed by “dental” (36 times), “dentistry” and “oral” (34 times). Word frequency in journal titles is graphically represented by the world cloud in Fig. 3. Words reflecting the worldwide character (e.g., “International”, “World”, “Global”) or the origin of the journal (e.g., American, Asian) was documented in 39 journals with “International” accounted for the 69.23% of the appealing titles.

**Table 3:** Examples of flattery used in the e-mail invitations examined in the study

“It is our honour to invite eminent scientists like you.”
“We really value your outstanding contribution to the scientific community.”
“Based on your eminent expertise and immense contributions in the field of …, we warmly solicit your participation in the upcoming issue.”
“As a leading expert in your field, we would like you to participate by submitting your research…”
“We are honoured to invite you to submit your valuable research work for publication in our Journal.”
“We came across your profile, and based on your work, we would be privileged if you could join us.”
“It is our pleasure to have your profile in our Journal Editorial Board.”
“Your dedication, enthusiasm, and insights are inspiring us and we would like to invite you to join our Editorial/Reviewer Board.”

## Discussion

This study aimed at measuring the prevalence and characteristics of predatory publishing in orthodontics in order to alert researchers for the most common strategies of illegitimate publishers and possible consequences for co-operating voluntarily or due to lack of knowledge with such journals. Based on our results, researchers in orthodontics should be aware that more than 7 out of 10 unsolicited electronic invitations for article contribution or participation as an editorial board member or reviewer may derive from journals suspicious for publishing malpractices and suboptimal standards. Overall, the aggressive promotional campaigns of predatory journals were confirmed for several journals. The number of invitations from each journal/publisher received within the 1-year observation ranged from 1 to 56. Like previously observed by Clemons et al., multiple e-mail invitations from different journals published by a single publisher were signed by the same editorial manager or assistant, pointing to the profit-driven tactics of those publishing houses [23].

### Journal characteristics

To stress the scientific performance and identity of the journals, e-mails from publishers included in this study provided sporadically IFs and ISSNs. The use of false or misleading metrics like journal impact factors, either made up or compiled by bogus companies, has been described as one of the salient features of predatory journals [2]. Though ISSN does not infer journal quality, use of such identifiers when combined with other parameters may indicate that the publisher follows guidelines promoting best journal publishing practices [24].

Editorial services such as peer-review and plagiarism check were mentioned in more than half of the solicitations. Several journals also claimed short manuscript turnaround times with accepted articles getting published within 2–3 weeks after submission. However, fast-paced publication procedures, though valuable in the dissemination of time-sensitive research when carried out properly, may question the rigor of the reviewing process. More than a few publishers in the present study recommended manuscripts to be submitted as e-mail attachment, probably skipping a number of essential quality checks performed otherwise automatically by online submission platforms. Manuscript submission to legitimate journals is generally managed via an online system that runs comprehensive quality control procedures to ensure, among others, adherence to authorship standards, declaration of conflicts of interest, compliance with word limit or absence of plagiarism before submission approval is granted [3].

Details on actual publication costs were occasionally present, and even when a full or partial APC waiver was offered, “DOI, designing or production charges” were applicable. In addition to this, submission deadlines were short and difficult to meet eliminating in this manner any claimed financial benefits. APCs in predatory journals have been estimated to be more than 10 times lower than in presumed legitimate, fully open access biomedical journals [3]. As predatory journals intend to attract as many submissions as possible to increase revenue, extremely low article publication or processing charges, even under $150, should raise a red flag about the legitimacy of the publishers [3]. In line with our findings, APCs as low as $50 have been reported for predatory journals elsewhere [25].

### E-mail language and contact details

Flattering or excessively flattering language about recipient’s skills and reputation in order to draw his attention was recorded in 42% of the solicitations. Common findings were also the abundance of grammatical errors, and the use of noisy layouts including mixed font sizes and types, colored, highlighted or underlined text, awkward sentence structure [12], and informal writing style indicating familiarity and non-scientific terms, all referring to lay language and not communication handled by the editorial offices of esteemed scholarly journals. Moreover, the use of appealing words and/or familiar journal titles intending to mislead less experienced researchers is well documented in the literature [2, 26]. Lack of efficient planning was also evident in several cases where the publishers claimed for a shortfall or urgency of papers [25] to facilitate “the successful release of the coming issue”, a scenario unlikely to occur in reputed scientific journals. USA was by far the most frequently purported country of origin as previously shown [11, 25]. Contact phone numbers and complete address details were not constantly provided while very often, only the state or city, where the journal/publisher headquarters was located, was available. Therefore, the results on the origin of journals/publishers may be treated in some cases with caution.

### E-mail relevance to recipient’s expertise

Failure to properly match the expertise of the e-mail’s recipient with the journal’s scope is routinely seen in predatory publishing invitations [13]. Like a previous dental study on predatory publishing [27], less than one-third of the journals were related to dentistry. A wide scope of interest combining non-biomedical and biomedical subjects has been reported as one of the main characteristics of presumed predatory journals [3]. Several non-biomedical journals dealt with a broad variety of disciplines, irrelevant to the invitee’s specialty or research interests. Only one invitation was received from an orthodontic journal, which was not included in the Beall’s and Cabell’s blocklists. It can be assumed that due to the relatively recent introduction of the journal, the editorial office might have attempted to inform on one occasion researchers in orthodontics about the opening of article submissions. Hypothetically, the relatively narrow scope of the orthodontic specialty and limited expected gains may discourage predatory publishers to launch orthodontic journals.

### Study limitations

This study is not without limitations. Dichotomous classification of journals as predatory or legitimate may not be straightforward. As the number and severity of violations of best practices may vary among journals, certain journals may be considered to operate in an undefined grey zone between fraud and legitimacy [15]. Non-inclusion of a journal in the blocklists at the time this study was conducted does not necessarily exclude predatory behavior. Due to the increasing number of new journal entries [7], it is likely that newly emerged journals might have not been reviewed yet by the administrators of the lists. It can be argued that examination of journal webpages could have provided more comprehensive overview of their publishing practices and standards and may therefore be investigated by future research on predatory publishing.

### Recommendations for tackling predatory publishing invitations

As a general rule, most researchers and faculty members are not formally trained on publication practices and ethics [3]. Providing useful guidance in managing journal selection and submission processes, in other words, “empowering authors with knowledge is an important step in decision-making” [28]. Additionally, academic career development is nowadays substantially centered on publication and funding record [14]. As long as the “publish or perish” culture continues to thrive in the academia, the list of predatory journals will be growing longer. Thus, academic institutions need to invest on educational resources and mentoring of young faculty to address unsolicited emails, disapprove publications in predatory journals in tenure and promotion decision making, and further refine IT infrastructure to block suspicious unsolicited emails [14]. Finally, the similarities observed across the available checklists suggest the creation of one evidence-based tool, applicable to all disciplines to assist scholars in spotting predatory and legitimate journals and publishers in scholarly communication [6].

## Conclusions

- An overload of e-mail unsolicited invitations for scholarly contribution was retrieved from an orthodontist’s inbox within a 12-month observation period.
- Nearly 8 out of 10 solicitations may be assumed to have originated from potential predatory journals.
- Typical features of predatory journals like flattering language and abundant grammatical errors in the e-mail invitations, and a wide variety of article topics and types invited for submission as well as incomplete journal contact details were observed in the present study.
- Researchers in orthodontics should be aware of predatory publishing practices and detrimental effects of collaboration with such journals and publishers on science and society.

## Data Availability

The datasets used and/or analyzed during the current study are available from the corresponding author on reasonable request.

## Abbreviations

OA: Open access
APCs: Article publication or processing charges
DOAJ: Directory of Open Access Journals
IF: Impact factor
ISSNs: International Standard Serial Numbers
DOIs: Digital object identifiers
